# Obesogenic environments and cardiovascular disease: a path analysis using US nationally representative data

**DOI:** 10.1186/s12889-022-13100-4

**Published:** 2022-04-10

**Authors:** Fangqi Guo, Georgiana Bostean, Vincent Berardi, Alfredo J. Velasquez, Jennifer W. Robinette

**Affiliations:** 1 Psychology Department, Crean College of Health and Behavioral Sciences, Chapman University, One University Drive, Orange, CA 92866 USA; 2 Department of Sociology, Humanities, and Social Sciences, Wilkinson College of Arts, Chapman University, Orange, CA USA; 3 Environmental Science & Policy Program, Schmid College of Science and Technology, Chapman University, Orange, CA USA

**Keywords:** Obesogenic environment, Cardiovascular disease, Mediation analysis, Health behaviors, Physiological dysregulation

## Abstract

**Introduction:**

People living in obesogenic environments, with limited access to healthful food outlets and exercise facilities, generally have poor health. Previous research suggests that behavioral risk factors and indicators of physiological functioning may mediate this link; however, no studies to date have had the requisite data to investigate multi-level behavioral and physiological risk factors simultaneously. The present study conducted serial and parallel mediation analyses to examine behavioral and physiological pathways explaining the association between environmental obesogenicity and cardiovascular disease (CVD).

**Methods:**

This cross-sectional observational study used data from the 2012–2016 Health and Retirement Study, a representative survey of US older adults (*n* = 12,482, mean age 65.9). Environmental obesogenicity was operationalized as a combined score consisting of nine environmental measures of food and physical activity. CVD and health-compromising behaviors (diet, alcohol consumption, smoking, and exercise) were self-reported. Physiological dysregulation was assessed with measured blood pressure, heart rate, HbA1c, cholesterol levels, BMI, and C-reactive protein. The Hayes Process Macro was used to examine serial and parallel paths through health-compromising behaviors and physiological dysregulation in the environmental obesogenicity-CVD link.

**Results:**

People living in more obesogenic environments had greater odds of self-reported CVD (odds ratio = 1.074, 95% confidence interval (CI): 1.028, 1.122), engaged in more health-compromising behaviors (β = 0.026, 95% CI: 0.008, 0.044), and had greater physiological dysregulation (β = 0.035, 95% CI: 0.017, 0.054). Combined, health-compromising behaviors and physiological dysregulation accounted for 7% of the total effects of environmental obesogenicity on CVD.

**Conclusion:**

Behavioral and physiological pathways partially explain the environmental obesogenicity-CVD association. Obesogenic environments may stymie the success of cardiovascular health-promotion programs by reducing access to resources supporting healthy lifestyles.

**Supplementary Information:**

The online version contains supplementary material available at 10.1186/s12889-022-13100-4.

## Background

Obesogenic environments with limited access to healthful food outlets and facilities for physical fitness are associated with increased cardiovascular disease (CVD) risk [1, 2]. Residents of obesogenic areas are more likely to smoke, consume excessive alcohol, be physically inactive, and consume an unhealthy diet [3, 4]. These residents also exhibit physiological dysregulation, such as high blood pressure, abnormal body mass index (BMI), and elevated glucose levels, which are associated with increased CVD risk [5, 6]. These behavioral and physiological factors typically cluster together [7–9], yet researchers rarely have the necessary data to investigate these multiple factors simultaneously. The current study investigated the extent to which health-compromising behaviors and physiological dysregulation explain the association between environmental obesogenicity and CVD. 

### CVD and individual-level risk factors

CVD has been the leading cause of death in the United States in recent decades, with tremendous health and economic burdens in the US and globally [6]. The most common types of CVD are coronary heart disease, heart failure, arrhythmia, and stroke [6]. CVD is caused by a combination of genetic, lifestyle, and environmental risk factors [6]. Most of these risk factors are modifiable. For example, CVD could be largely prevented by maintaining a healthy lifestyle, including not smoking, abstaining from or consuming only moderate amounts of alcohol, eating a healthy diet, and engaging regularly in moderate physical activity [10]. In addition, many of these health behaviors co-occur [7]. A US survey reported that 52% of participants had two or more health-compromising behaviors, including physical inactivity, smoking, heavy drinking, and having overweight [11]. Díaz-Gutiérrez and colleagues quantified the co-occurrence of these health-compromising behaviors (including smoking, physical activity, diet, alcohol intake, television exposure, and nap habit), and found that individuals who engaged in fewer health-promoting behaviors have higher CVD risk [5].

In addition to behaviors, some physiological indicators are associated with elevated risk of CVD. Multi-system physiological dysregulation is a sub-clinical state that occurs when long-term stressors accumulate over the life course and disrupt the regulation of multiple physiological systems [12]. The indicators for physiological dysregulation—including BMI, blood pressure, cholesterol, Hemoglobin A1c (HbA1c), and C-reactive Protein (CRP)—may predict future risk of CVD and poor health [13, 14]. For example, among older adults, higher total cholesterol, blood pressure, or BMI is associated with higher CVD risk [15]. As with health behaviors, biological CVD risk factors tend to co-occur [9]. A retrospective study of older adults found that 34% of participants presented with three or more biological CVD risk factors [8]. As the number of biological risk factors with values in risky ranges increases, so does CVD risk [14].

### CVD and obesogenic environments

The risk of CVD is influenced not only by individual-level risk factors, but also by the broader environment in which individuals live. Obesogenic environments, defined as “the sum of influences that surroundings, opportunities, or conditions of life have on promoting obesity in individuals or a population [16],” are associated with increased risk of CVD among the residents [17]. For example, people living in neighborhoods with reduced access to grocery stores and supermarkets [1], and a higher density of fast food restaurants [1, 2, 18, 19] and convenience stores [20], have a higher risk of myocardial infarction, coronary heart disease, congestive heart failure, and stroke. Additionally, reduced access to parks and recreation facilities increases the odds of these same conditions [1, 18, 20].

It appears that obesogenic environments may increase CVD risk through their associations with health-compromising behaviors and physiological dysregulation. First, obesogenic environments are linked to health-related behaviors such as diet and physical activity [2]. These health behaviors are, in turn, associated with CVD risk [5]. For example, access to fast food restaurants and convenience stores is associated with excessive consumption of sugar-sweetened beverages and insufficient consumption of whole grains and vegetables [4, 21]. Conversely, closer proximity to supermarkets is linked to lower fat consumption [3] and greater consumption of fruits and vegetables [22]. Moreover, limited access to recreation facilities such as gyms and parks is associated with physical inactivity [23].

Furthermore, living in economically disadvantaged neighborhoods is associated with limited healthful food and physical activity resources which is, in turn, associated with increased alcohol and cigarette consumption [24]. Well-established conceptual frameworks assist with understanding this association. First, people living in economically disadvantaged neighborhoods have a higher risk of psychological distress [25]. Second, suggested by the tension reduction hypothesis, alcohol and cigarette consumption help people reduce stress [26]. It is therefore plausible that individuals living in obesogenic environments consume alcohol and tobacco products both because of their relative availability and as a means to relieve stress, and these compromising behaviors are associated with increased risk for CVD [6].

Additionally, residents living in obesogenic environments exhibit elevated physiological dysregulation [2]. For example, people living in neighborhoods with greater access to convenience stores or fast food restaurants, and those with limited access to grocery stores or farmers markets have higher BMI [2, 27], greater hypertension risk [2], higher HbA1c, and increased blood glucose levels, which indicate increased risk of Type 2 diabetes [2, 28]. Proximity to supermarkets is associated with lower prevalence of obesity and overweight; conversely, the presence of convenience stores is associated with a higher prevalence of overweight and obesity [29]. Moreover, environments with fewer facilities for physical fitness are associated with higher BMI and higher prevalence of hypertension [30]. Thus, the association between environmental obesogenicity and physiological dysregulation may be due to engaging in health-compromising behaviors, such as maintaining an unhealthy diet [4].

### Current study: identifying pathways linking obesogenic environments and CVD

The complex interrelationships among individual-level and environmental-level risk factors and CVD suggest at least two potential pathways linking environmental obesogenicity and CVD [31]. The behavioral pathway suggests that limited access to healthy foods and facilities for physical fitness predict poor diet [32] and inadequate physical activity [23]. The physiological pathway suggests that living in obesogenic environments may contribute to the gradual deterioration of human physiological regulatory systems, perhaps through stress-related or psychosocial processes [12]. An alternative plausible conceptualization is serial mediation, in which the accumulation of health-compromising behaviors predict the early precursors of physiological dysregulation, which in turn, relates to cardiovascular health [4]. Combined, these behavioral and physiological processes may increase residents’ risk for CVD.

**Fig. 1:**
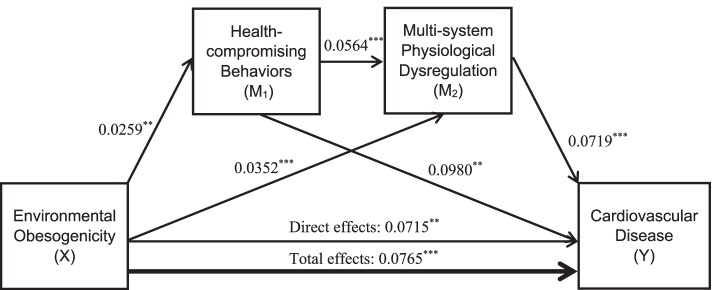
Conceptual Model; Path Diagram Illustrating Direct and Indirect Effects Linking Environmental Obesogenicity and Cardiovascular Disease. Note: Environmental Obesogenicity (X) incorporates number of grocery stores, farmer’s markets, superstores, recreation centers, convenient stores, fast food restaurants, and no access to a private vehicle and in an area with low access to a healthful food store per 1000 residents, and crime rates. Health-compromising behaviors (M_1_) consist of diet, alcohol use, smoking, and physical activity. Multi-system physiological dysregulation (M_2_) incorporates blood pressure, heart rate, hemoglobin A1c, total cholesterol, high-density lipoprotein cholesterol, BMI, and C-reactive protein. Covariates (age, sex, highest degree, race/ethnicity, county-level education, and county-level population density) were adjusted in all analyses. Numbers indicate coefficients of each pathway. ^*^p < 0.05; ^**^p < 0.01; ^***^p < 0.001

Despite evidence that various aspects of the environment are related to health behaviors and physiological functioning, few studies have investigated multiple behavioral and physiological pathways through which residential environments are linked to residents’ cardiovascular health. This study uses a representative sample of US adults aged 51 and over, a group of individuals who may be less mobile than younger adults and rely more heavily on neighborhood resources [33], to test whether the association between environmental obesogenicity and CVD can be explained by health-compromising behaviors or multi-system physiological dysregulation as parallel pathways, or if these factors operate as serial mediators (see Fig. 1). We hypothesized that the association between environmental obesogenicity and CVD was partially explained by 1) health-compromising behaviors, 2) physiological dysregulation, both individually and 3) in serial order (environment health behaviors physiological dysregulation CVD).

## Methods

### Data

To test the hypotheses, we conducted a cross-sectional observational study using data from the Health and Retirement Study (HRS), an ongoing longitudinal survey of more than 20,000 US older adults [34]. The target population for HRS includes all older adults (≥ 51) living in the contiguous United States (including 48 States and the District of Columbia). The HRS used multi-stage area probability sample design to recruit participants to ensure the representativeness of the sample [35]. In 1992, HRS began collecting data on health conditions, health behaviors, and socioeconomic circumstances associated with aging. Since HRS inception, refresher cohorts have been recruited to include representative samples of the Asset and Health Dynamics (1993), Children of the Depression (1998), War Babies (1998), Early Baby Boomers (2004), Mid Baby Boomers (2010), and Late Baby Boomers (2016) [34]. After the initial 1992 interview, the participants were interviewed every two years. The response rate of each wave was between 80%—90% [36]. Since 2006, HRS has expanded data collection to include blood, saliva, and anthropometric measures as a part of enhanced face-to-face (EFTF) interviews [36]. Half of the sample was randomly selected to complete the EFTF in 2006, and the other half completed it in 2008. Both half-samples repeat the EFTF every four years. In 2013, HRS conducted a Health Care and Nutrition Mail Study (HCNS) in which HRS researchers mailed the Harvard food frequency questionnaire (FFQ) to a subsample of HRS respondents. HRS health records were linked to a Contextual Data Resource (CDR) including the USDA Food Environment Atlas [37], the US Uniform Crime Reporting Program [38], and the American Community Survey data sets via geographic identifiers [39].

The analytical sample of the current study included participants from all 48 contiguous states and the District of Columbia residing in 675 counties. The characteristics of the full study sample and subsamples stratified by tertiles of environmental obesogenicity are displayed in Table 4.

### Measures

#### Environmental obesogenicity (Key independent variable)

Our environmental obesogenicity index (see Table 1) was constructed from nine indicators reflecting food, physical activity, social-structural, and economic environments between 2009 and 2012. Of the nine indicators, five were considered as salubrious resources, including county-level counts of grocery stores, farmers markets, superstores, and recreation centers per 1000 residents, as well as county-level median family income. The remaining four health-compromising indicators included county-level counts of convenience stores, and fast food restaurants per 1000 residents, as well as crime rates, and census tract-level proportion of residents with no vehicle and low access to a healthful food store. The definition of low access to a healthy food store was based on distance (> 0.5 miles for urban areas, or > 10 miles for rural areas) from a supermarket, supercenter, or grocery store. Crime rates were the sum of murder, rape, robbery, aggravated assault, burglary, larceny, auto theft, and arson, adjusted for total county population. Our measurement of environmental obesogenicity, based on the Childhood Obesogenic Environment Index (COEI) [40], was constructed mainly of county-level indices (mean, range US counties: 3,130 km^2^, [0.026–376, 869 km^2^]). Although county-level measures may not capture the nuance of individuals’ daily activity spaces which requires more geographically granular data, they are associated with health outcomes [41]. In addition, the present study was conducted to investigate relationships between access to healthful food and fitness facilities and health, and residents generally don’t access these resources from their neighborhood block [42].

**Table 1:**
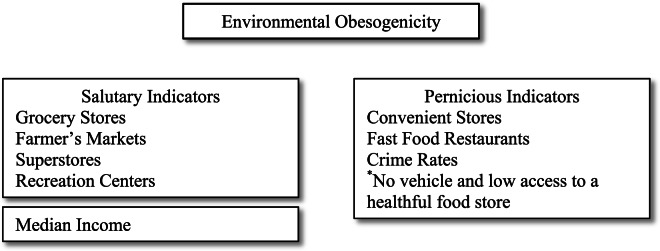
Environmental Obesogenicity Index (Scale 0—100)

Each indicator represents county-level counts per 1000 residents with the exception of a census tract-level variable for no vehicle and low access to a healthful food store

^*^Census tract-level variable

To guide the construction of our scale, we used both the COEI which defines access to healthful food by count of outlets [40], and the USDA, which defines access to healthful food by distance to outlets and vehicle ownership [37]. Following the USDA definitions, we defined access to healthful food markets differently for rural and urban areas, due to population density differences; a distance of < 0.5 miles in urban areas was considered to be accessible, within 10 miles in rural areas [37].

For each indicator, all counties/tracts were ranked and assigned a percentile. Salubrious environmental variables were reverse-scored, such that higher values on the final score indicated greater neighborhood obesogenicity. The final environmental obesogenicity index was constructed by averaging across the nine percentile-ranked indicators. The range of the index for all US census tracts was 20–93. The index for the study participants ranged from 22–82 (see Table 4), suggesting that the census tracts represented in the analytic sample represent nearly the full range of possible scores in the US. All variables used for the index came from the USDA food environment and access data file V2.0, with the exception of crime rate from the Uniform crime reporting program data V2.0 and total county population from the American Community Survey (ACS) 2008 – 2012 five-year estimates. The external data files were linked to HRS participant records using geographic identifiers.

#### Presence or absence of CVD (Dependent variable)

Study participants self-reported in 2016 whether they had ever been diagnosed with any of the following cardiovascular diseases: heart attack, coronary heart disease, angina, congestive heart failure, or other heart problems. Participants with at least one type of CVD were coded as 1, and participants with no CVD were coded as 0. About one-quarter (23%) of study participants self-reported having at least one CVD (see Table 4).

#### Health-compromising behaviors (Behavioral mediator)

Since many health behaviors co-occur [7], researchers have attempted to quantify the constellation of behaviors in which individuals engage by constructing composite lifestyle indices that classify people’s behaviors as health-compromising or health-promoting [5, 43]. Our index of health-compromising behaviors (see Table 2) was based on the method published by Tabung and colleagues [43]. The index for the current study was comprised of four behaviors including smoking, alcohol consumption, exercise frequency, and diet. The first three of these behavioral indicators came from 2012 HRS Core interview and the RAND file v2.0 [44]. Participants were asked about their smoking status (1 = current smoker, 0.5 = ever smoked, 0 = never smoked). Alcohol consumption was assessed with the question “In the last three months, on the days you drink, about how many drinks do you have”. Women who reported having more than 1 drink per day, and men who reported having more than 2 drinks per day were identified as engaging in excessive drinking behavior (1), with all others classified as engaging in low/moderate drinking (0). Participants engaging in moderate (e.g., gardening, walking at a moderate pace, dancing, or stretching), or vigorous (e.g., running, jogging, or swimming) activities more than once a week were identified as physically active (0); all others were identified as physically inactive (1).

**Table 2:** Health-compromising Behavior Index (Scale 0–4)

Indicators and cutoff points
Smoking status (1 = current smoker, 0.5 = ever smoked, 0 = never smoked)
Drinking
1 = excessive drinking (> 1 drink per day for women or > 2 drinks per day for men)
0 = moderate or no drinking (≤ 1 drink per day for women or ≤ 2 drinks per day for men)
Physical activity
1 = physical inactive (engage in moderate, or vigorous activities more than once a week)
0 = physical active (engage in moderate, or vigorous activities once a week or fewer)
Diet
1 = unhealthy diet (violate two or more recommended limits listed below)
0 = healthy diet (violate fewer than two recommended limits listed below)
Daily nutrition intake recommended by USDA dietary guideline (2015):
added sugar < 10% total daily calories
sodium < 2300 mg per day
saturated fat < 10% total daily calories
cholesterol < 300 mg per day
fiber ≥ 28 g per day for men or ≥ 22.4 g per day for women

Diet behavior was constructed with data from the HCNS sub-study (*n* = 8,073) in 2013 [34]. CDC and USDA both recommend diets with low sugar (< 10% of total daily calories), low sodium (< 2300 mg per day), low saturated fat (< 10% of total daily calories), low cholesterol (< 300 mg per day), and adequate fiber (≥ 28 g fiber per day for men or ≥ 22.4 g fiber per day for women age 59 and older) to lower CVD risk [45, 46]. Participants in the HCNS received a questionnaire to report the consumption of food and beverages. Participants whose self-reported consumption violated two or more recommended limits were identified as engaging in unhealthy dietary behaviors [1], with all others classified as engaging in healthy dietary behaviors (0). A final index of health-compromising behaviors was constructed by summing across the four dichotomous variables (range 0–4), with higher values indicating more health-compromising behaviors. On average, study participants engaged in 1.2 health-compromising behaviors (see Table 4). Supplemental Table 1 displays how the combined behavioral score was constructed.

#### Multi-system physiological dysregulation (Physiological Mediator)

An index of physiological dysregulation representing functioning across multiple regulatory systems was constructed for the purposes of this study (see Table 3) [14]. The choice of indicators was guided by prior research and data availability [13]. The indicators included were systolic blood pressure (SBP), diastolic blood pressures (DBP), and heart rate (indices of cardiovascular health); hemoglobin A1c, total cholesterol (TC), high-density lipoprotein cholesterol (HDL), BMI (indices of metabolism), and C-reactive protein (an index of immune system). All variables were from 2014 and 2016 HRS half-samples, combined in this study for a complete sample. SBP, DBP, and heart rate were the average of three measurements. BMI was calculated from measured weight and height. All other indicators were assessed with dried blood samples.

**Table 3:** Indicators and ‘At Risk’ Values of Physiological Dysregulation Index (Scale 0—8)

Indicator	Physiological system	Clinical cut-point
Systolic blood pressure (SBP)	Cardiovascular system	≥ 140 mmHg
Diastolic blood pressure (DBP)	Cardiovascular system	≥ 90 mmHg
Heart rate	Cardiovascular system	≥ 90 beats/min
Hemoglobin A1c	Metabolism	≥ 6.5%
Total cholesterol (TC)	Metabolism	≥ 240 mg/dl
High-density lipoprotein cholesterol (HDL)	Metabolism	< 40 mg/dl
Body mass index (BMI)	Metabolism	≥ 25 (overweight) or < 18.5 (underweight)
C-reactive protein	Immune	≥ 3.0 mg/dl

Each indicator was dichotomized as ‘not risky’ (0) or ‘risky’ (1) based on its clinical cut-points. The thresholds for the risky categories were: SBP ≥ 140 mmHg, DBP ≥ 90 mmHg, heart rate ≥ 90 beats/min, A1c ≥ 6.5%, TC ≥ 240 mg/dl, HDL < 40 mg/dl, BMI ≥ 25 or < 18.5, and C-reactive protein ≥ 3.0 mg/dl. The physiological dysregulation index was defined as the sum of indicators in the risky category, ranging from 0–8, with higher scores indicating greater physiological dysregulation. On average, study participants had 1.9 physiological indicators with values above clinical cut-points, indicating dysregulation (see Table 4).

**Table 4:** Characteristics of the full study sample, and subsamples stratified by tertiles of environmental obesogenicity: Health and Retirement Study, United States, 2012 – 2016

Variables	Mean ± SD (min – max) or percent (%)			
	Full sample	Low-obeogenicity	Moderate-obesogenicity	High-obesogenicity
N	12,482	4245	4121	4116
Age (years)	65.9 ± 9.8 (51 – 102)	65.5 ± 9.9 (51 – 99)	65.9 ± 9.8 (51 – 15)	66.2 ± 9.6 (51 – 102)
Sex				
Male	41.0%	42.6%	42.0%	38.2%
Female	59.0%	57.4%	58.0%	61.8%
Race and ethnicity				
Non-Hispanic White	63.7%	73.8%	65.6%	51.5%
Non-Hispanic Black	19.3%	11.7%	17.5%	29.0%
Hispanic	8.7%	5.7%	8.9%	11.5%
Other	8.3%	8.8%	7.9%	8.0%
Highest degree of education				
No degree	17.2%	12.9%	14.7%	24.0%
High school diploma or GED	52.6%	51.2%	54.0%	52.7%
College degree	20.0%	23.3%	20.3%	16.4%
Graduate degree	10.1%	12.5%	11.0%	6.9%
Environmental obesogenicity	49.0 ± 9.5 (22.3 – 82.1)	39.3 ± 4.4 (22.3 – 44.6)	48.4 ± 2.3 (44.7 – 52.3)	59.7 ± 6.3 (52.4 – 82.1)
Self-reported cardiovascular disease				
Presence	73.0%	75.2%	72.5%	71.2%
Absence	27.0%	24.8%	27.5%	28.8%
Health-compromising behaviors	1.2 ± 0.9 (0 – 4)	1.1 ± 0.9 (0 – 4)	1.1 ± 0.9 (0 – 4)	1.2 ± 0.9 (0 – 4)
Physiological dysregulation	1.9 ± 1.2 (0 – 7)	1.8 ± 1.2 (0 – 6)	1.9 ± 1.2 (0 – 7)	2.1 ± 1.3 (0 – 7)

*Abbreviations*: *SD* standard deviation, *min* minimum, *max* maximum, *GED* general education development

#### Covariates

Baseline age in years (2012), sex (male, female), race/ethnicity (non-Hispanic White, non-Hispanic Black, Hispanic, and Other), and highest degree (no degree, high school diploma/general education development, college degree, and graduate degree) were included as covariates given their association with both living environment and CVD [6, 47]. Since the predictor of the present study (environmental obesogenicity) is assessed using county and tract-level variables, two contextual variables were drawn from the American Community Survey 2008–2012 five-year estimates and used as covariates: percent of high school graduates (25 years old and over) with no college degree, and population density per square mile. County-level degree is an indicator of socioeconomic status, which is selected to match with individual-level degree; population density is included because of its association with environmental obesogenicity [4].

### Statistical analysis

All analyses were conducted in SAS 9.4 using the Hayes Process Macro V2.13, a path analysis modeling tool that uses ordinary least squares (OLS) and logistic regressions for estimating direct and indirect effects in parallel and serial mediation models [48]. We used Process Macro Model 6 to conduct parallel and serial mediation analyses to test the extent to which health-compromising behaviors, physiological dysregulation, or both in serial order (environment → health behaviors → physiological dysregulation → CVD) explain the hypothesized relationship between environmental obesogenicity and CVD. The current study examined serial mediation, in which health-compromising behaviors preceded physiological dysfunction. We also examined a reverse mediation pathway (obesogenicity → dysfunction → behaviors → CVD) since people with physiological dysregulation may tend to be more physically inactive (see Supplemental Table 3). Results indicated that compared to parallel mediation pathways, both serial mediation pathways explained a negligible portion of the total effects (< 0.3%). As such, and because individuals' behaviors generally don't change substantially after physiological complications [49], we only report results of our originally hypothesized model in which health-compromising behaviors preceded physiological dysregulation.

Individual-level age, sex, education, race/ethnicity, and county-level education and population density were included as controls. Continuous variables were standardized prior to mediation analysis. Coefficients presented in results are therefore partially standardized regression coefficients (B). Observations with any missing data in analytical variables were excluded from the analyses, leaving a final sample size of 12,482. To avoid temporal confounding, we employed a fully lagged mediation design, i.e., the independent variable neighborhood obesogenicity contained indices available between 2009 and 2012; the outcome variable CVD was assessed in 2016; the two mediators – health-compromising behaviors and physiological dysregulation – were measured in 2012–2013 and 2014–2016, respectively. As such, we prioritized a temporal schema whereby the predictor preceded the mediators, which in turn, preceded the outcome. In other words, contemporaneous effects between mediators and outcomes were not permitted. Data sources and available years of each indicator are presented in Supplemental Table 2.

## Results

Characteristics of the full study sample and subsamples stratified by tertiles of environmental obesogenicity are displayed in Table 4. Of the 12,482 study participants (mean age 65.9 years, range 51—102), 59.0% are women, 41.0% are men, 63.7% are non-Hispanic Whites, 19.3% are non-Hispanic Blacks, 8.7% are Hispanics, and 8.3% are Others.

In support of our hypotheses, the results of parallel and serial mediation analyses (Table 5) indicated that people living in more obesogenic neighborhoods had greater log odds of reporting CVD than those living in less obesogenic environments (total effects = 0.0765, 95% Confidence Interval (*CI):* 0.0328, 0.1202). The two mediators, health-compromising behaviors and physiological dysregulation, explained 7% of the total effects of environmental obesogenicity on CVD (combined indirect effects = 0.0052, 95% *CI*: 0.0026, 0.0085). This left 93% of the environmental obesogenicity-CVD relationship unaccounted for by the two mediators (direct effects = 0.0715, 95% *CI*: 0.0278, 0.1153). Of the total indirect effects of environmental obesogenicity on CVD, 48% was through health-compromising behaviors, another 48% was explained through physiological dysregulation, and a very small portion (2%) of indirect effects were through the serial pathway from health-compromising behaviors to physiological dysregulation and finally to CVD risk.

**Table 5:** Total Effects, Direct Effects, and Indirect Effects Between Environmental Obesogenicity and CVD (*n* = 12,482)

Path	Coefficient^a^	Boot LLCI	Boot ULCI
Total effects of X on Y	0.0765	0.0328	0.1202
Direct effects of X on Y	0.0715	0.0278	0.1153
Indirect effects of X on Y	0.0052	0.0026	0.0085
* through M*_*1*_	0.0025	0.0007	0.0052
* through M*_*2*_	0.0025	0.0009	0.0050
* serially through M*_*1*_* and M*_*2*_	0.0001	0.0000	0.0002
Effects through mediators			
X → M_1_	0.0259	0.0075	0.0444
X → M_2_	0.0352	0.0167	0.0537
M_1_ → Y	0.0980	0.0561	0.1399
M_2_ → Y	0.0719	0.0302	0.1136
M_1_ → M_2_	0.0564	0.0388	0.0740

Age, sex, highest degree, race/ethnicity, county-level education, and county-level population density were adjusted in the analysis

^a^X, M_1_, M_2_ were standardized prior to analysis

Boot LLCI, bootstrapped lower limit confidence interval

Boot ULCI, bootstrapped upper limit confidence interval

X, environmental obesogenicity

Y, presence of self-reported CVD

M_1_, health-compromising behaviors

M_2_, multi-system physiological dysregulation

Further decomposition of the effects through both mediators indicated that people living in more obesogenic environments engaged in significantly more health-compromising behaviors (coefficient (β) = 0.0259, 95% *CI: 0.0075, 0.0444*) and had greater physiological dysregulation (β = 0.0352, 95% *CI: 0.0167, 0.0537*). People engaging in more health-compromising behaviors (β = 0.0980, 95% *CI: 0.0561, 0.1399*) and those with greater physiological dysregulation (β = 0.0719, 95% *CI:0.0302, 0.1136*) had greater log odds of reporting at least one CVD (see Fig. 1).

Regarding the study covariates (Table 6), lower odds of reporting CVD were observed among participants with relatively younger age, women (compared to men), Hispanics (compared to non-Hispanic Whites), and those with college degrees (compared to no degree). Further, people living in counties with higher percentages of college degree-holders had lower log odds of reporting CVD. County-level population density was not significantly associated with CVD.

**Table 6:** Effects of Environmental Obesogenicity, Health-compromising behaviors, Physiological Dysregulation, and Covariates on CVD (*n* = 12,482)

Variable	Odds Ratio	95% Confidence Interval
Environmental obesogencity^*^	1.074	1.028 – 1.122
Health-compromising behaviors^*^	1.103	1.058 – 1.150
Physiological dysregulation^*^	1.075	1.031 – 1.120
Age (in years) ^*^	1.663	1.593 – 1.735
Female^a^	0.731	0.673 – 0.795
Race/ethnicity^b^		
Non-Hispanic Black	0.908	0.807 – 1.023
Hispanic	0.712	0.597 – 0.848
Other	0.807	0.678 – 0.960
Degree^c^		
High school diploma or GED	1.006	0.894 – 1.132
College degree	0.861	0.744 – 0.996
Graduate degree	0.889	0.746 – 1.059
County-level degree^*d^	1.080	1.029 – 1.134
County-level Population density^*^	1.022	0.974 – 1.072

^*^Variables were standardized prior to analysis

^*a*^*Compared to male*

^*b*^*Compared to Non-Hispanic White*

^*c*^*Compared to No degree*

^*d*^*County-level percent of high school graduates with no college degree of population age 25* +

In order to address a potential bias related to residential mobility between the long study period (2009 – 2016), we conducted a sensitivity analysis, in which only individuals who did not move from 2009—2016 were included (*n* = 7,746). Study results suggested that there was still a significant association between environmental obesogenicity and CVD (total effects = 0.0691, 95% *CI*: 0.0135, 0.1248), as well as significant indirect effects through health-compromising behaviors and physiological dysregulation, combined (indirect effects = 0.0043, 95% *CI*: 0.0013, 0.0084).

## Discussion

This study investigated the behavioral and physiological pathways explaining the association between environmental obesogenicity and CVD. It was among the first, to our knowledge, to examine numerous aspects of obesogenic environments and to compare two conceptualizations of the mediating effects (parallel versus serial mediation) of health-compromising behaviors and physiological dysregulation in the association between environment and CVD. Our major finding was that these individual-level processes explained seven percent of the association between living in an obesogenic neighborhood and CVD. Moreover, parallel and serial mediation results supported that health-compromising behaviors and physiological dysregulation are better conceptualized as parallel rather than serial mediators. These findings were observed among a representative US sample of older adults, net of individual and county-level controls. Below, we discuss the main findings and their implications for public health.

### Environmental obesogenicity and CVD: investigating multiple pathways

Consistent with previous studies [1, 2, 18, 20], we found that people living in more obesogenic environments had greater odds of reporting CVD. However, we went beyond existing research by assessing environmental obesogenicity using nine indicators to capture the multidimensional nature and complexity of peoples’ environments. Combined, this collection of environmental indicators helped to partially explain the commonly co-occurring behavioral and physiological risk factors observed among residents of these environments.

Moreover, we extended the literature by demonstrating that both health behaviors and physiological dysregulation are implicated in this association. Our study examined a wider array of variables capturing a multitude of health-compromising behaviors and aspects of physiological dysregulation to better represent these commonly comorbid factors [7–9], whereas most prior studies typically included a single health-related behavior [31] or one biomarker [50]. We observed that residents of obesogenic environments engaged in more health-compromising behaviors [2, 4, 22, 23, 29, 51] and had greater physiological dysregulation [2, 3, 27, 28, 30] than those living in less obesogenic environments. Both behaviors and physiological dysregulation, in turn, were associated with CVD. While most previous studies did not formally test indirect effects and did not assess whether a significant proportion of variance was explained [2, 31], this study used path analysis to estimate the total, direct, and indirect effects through each pathway and the significance of each. We found that health-compromising behaviors and physiological dysregulation each explained similar proportions of the total effect (3.3% vs 3.3%), indicating that both health-compromising behaviors and physiological dysregulation partially explain the obesogenicity-CVD link. This was comparable to the study by Yang et al., which found that physiological risk factors explained between 3 and 11% (depending on the physiological measure) of the link between residential greenness and CVD risk [50]. Although our study examined obesogenicity rather than greenness, our findings are generally consistent with those of Yang et al., that physiological risk factors explained a small portion of the environment-CVD link. It is plausible that other potential pathways, though not the focus of the current study, may also explain the obesogenicity-CVD relation, such as stress [25]. The purpose of the current study was to investigate specific mediators that would inform the development of targeted interventions to reduce CVD, but not to identify a model that completely explains the obesogenic environment – CVD relationship. Future research should investigate other potential pathways that contribute to the relationship.

Finally, results suggested that behavioral and physiological factors each partially, both in tandem and – to a much smaller degree – in serial order, explained the significant association between environmental obesogenicity and CVD. This finding made a major contribution to the literature by enhancing scholars’ understanding of the conceptual links between these factors. Although conventional logic suggested that behaviors led to physiological dysregulation which affected health, our findings showed that this serial mediation conceptualization was not a substantially better model for these associations. That is, physiological dysregulation may also operate as a separate mechanism from behaviors (potentially through other pathways such as stress). It is plausible that the time lag between our measures is not sufficiently long enough to capture physiological changes resulting from behaviors, and future research should continue to compare alternative conceptualizations of these associations, as well as additional pathways linking environment and CVD.

### Limitations

Results should be interpreted with several limitations in mind. First, although we modeled the environmental obesogenicity measure after the COEI [40], data on walkability and access to parks which were included by COEI, are not available in the HRS. However, based on the USDA Food Access Research Atlas [37], our measure of environmental obesogenicity included two additional variables: county-level median household income and census tract-level proportion of residents with low-access to healthful food stores, which combined urban/rural-adjusted distance to the closest healthful food venue and average vehicle access. The former was an indicator of area-level food and physical activity resources, and the latter was used to define low access to healthy food outlets by the USDA Food Access Research Atlas [37]. The present study extended COEI to a representative older population with a wider array of mediators and outcomes. Second, the dietary information available in HRS came from a non-representative subsample of participants in the HRS HCNS. In our final analytical sample, 5,557 people had dietary information, so the health-compromising behavioral index was assessed without diet information from many participants in this study (summed across smoking, drinking, and physical activity). Third, to test the serial mediation pathway, we employed a fully lagged mediation design, i.e., the measure of environmental obesogenicity preceded two mediators, which preceded the measure of CVD (see Supplemental Table 2 for a description of data availability across HRS waves of data collection). This lagged mediation design, however, introduced a potential bias related to residential mobility between the long study period. However, based on the results of the sensitivity analysis, which only included people who did not move during the study period (*n* = 7,746), there was still a significant association between environmental obesogenicity and CVD, and significant indirect effects through health-compromising behaviors and physiological dysregulation, combined. Furthermore, CVD and health-compromising behaviors were self-reported in the present study, inviting bias related to several factors including memory, social desirability, and perhaps most importantly, variability in access to regular care. However, we believe that concern regarding self-report bias is somewhat minimized given that the current study found a significant association between the objectively measured physiological dysregulation composite variable and self-reported CVD. Indeed, this finding supports the future use of self-reported CVD data in instances when more objective physician reports are unavailable. Moreover, we found a modest effect of neighborhood environment on health, consistent with research showing that neighborhood effects on health are typically small [52]. Last, study variables used in the present study were at multiple levels, i.e., predictors were at county/tract level, mediators and outcomes were at individual level, and covariates were at both levels. Including variables at different levels of aggregation may lead to the modifiable areal unit problem [53], which may introduce challenges to interpretation of the findings. Despite these limitations, this study went beyond existing research in the robustness of measures and statistical methods used, providing valuable insight into the roles of health behaviors and physiological dysregulation in the obesogenicity-CVD association.

## Conclusion

The present study found that people who were living in obesogenic environments had both greater engagement in health-compromising behaviors and greater physiological dysregulation. Both of these behavioral and physiological processes are known to increase CVD risk [5, 14], which the findings of the current study support. Our results suggest that one strategy that local government may implement to decrease residents’ risk for CVD is decreasing the obesogenicity of the area by encouraging development of more healthful food outlets and/or recreation centers. More importantly, the effectiveness of individual-level prevention strategies that target these behavioral and physiological processes may be substantially reduced in settings where area-level resources are constrained. Therefore, addressing the risk of environmental obesogenicity may be the first step in developing cardiovascular health-promotion programs. Moreover, results of the present study suggest that such cardiovascular health-promotion programs should address multiple individual-level behavioral and physiological processes simultaneously, for example by promoting healthy diet and physical activity, mastering stress management strategies, as well as maintaining proper weight and normal blood pressure.

## Supplementary Information

**Additional file 1:** **Supplemental Table 1.** Distribution of health-compromising behaviors (HCB) (*n* = 12,482). **Supplemental Table 2.** Data sources and available years of core study variables. **Supplemental Table 3. **Reversed serial mediation analysis (obesogenicity → dysfunction → behaviors → CVD) using data from 2010 – 2014 (*n* = 12,317).

## Data Availability

The current study used data from the Health and Retirement Study (HRS), which were collected by the University of Michigan. Most of the data that were used in the current study are publicly available via the HRS website (https://hrs.isr.umich.edu). Some data are restricted, which can be accessed through the virtual desktop infrastructure (VDI) system. Individuals who want to access the HRS restricted data will need to submit a form to apply for a Restricted Data Agreement (RDA). More details about applying for access can be found on the HRS website (https://hrs.isr.umich.edu/data-products/restricted-data/vdi).

## Abbreviations

CVD: Cardiovascular Disease
HbA1c: Hemoglobin A1c
CRP: C-Reactive Protein
BMI: Body Mass Index
SBP: Systolic Blood Pressure
DBP: Diastolic Blood Pressure
TC: Total Cholesterol
HDL: High-Density Lipoprotein Cholesterol
COEI: Childhood Obesogenic Environment Index
HRS: Health Retirement Study
ACS: American Community Survey
EFTF: Enhanced Face-To-Face Interviews.
HCNS: Health Care and Nutrition Mail Study
FFQ: Food Frequency Questionnaire
CDR: Contextual Data Resource
