# Supplementary material for: Obesogenic environments and cardiovascular disease: a path analysis using US nationally representative data
Source: BMC Public Health. 2022 Apr 10;22:703. doi: 10.1186/s12889-022-13100-4 (PMC8994874; doi:10.1186/s12889-022-13100-4)
Supplement: Supplementary file 1 — Additional file 1: Supplemental Table 1. Distribution of health-compromising behaviors (HCB) (n = 12,482). Supplemental Table 2. Data sources and available years of core study variables. Supplemental Table 3. Reversed serial mediation analysis (obesogenicity → dysfunction → behaviors → CVD) using data from 2010 – 2014 (n = 12,317). [file 12889_2022_13100_MOESM1_ESM.docx]

**Supplementary Information Files**

Supplemental Table 1. Distribution of health-compromising behaviors (HCB) (n = 12,482)

| HCB | Yes | No | n |
| --- | --- | --- | --- |
| Smoking | 13.8% (current smoker)  41.4% (previous smoker) | 44.83% | 12421 |
| Drinking alcohol | 15.9% | 84.0% | 12482 |
| Physical inactive | 43.3% | 56.7% | 12478 |
| Unhealthy diet | 52.4% | 47.6% | 5557 |

Supplemental Table 2. Data sources and available years of core study variables.

| Indicators | Data source | Measured Year | Used for this study |
| --- | --- | --- | --- |
| **Environmental Obesogenicity Index** | | | |
| Farmer’s market | USDA Food Environment Atlas by County | 2009, 2013, 2016 | 2009 |
| Grocery store | USDA Food Environment Atlas by County | 2007, 2009, 2012, 2014 | 2012 |
| Super center | USDA Food Environment Atlas by County | 2007, 2009, 2012, 2014 | 2012 |
| Fastfood restaurant | USDA Food Environment Atlas by County | 2007, 2009, 2012, 2014 | 2012 |
| Convenience store | USDA Food Environment Atlas by County | 2007, 2009, 2012, 2014 | 2012 |
| Recreation center | USDA Food Environment Atlas by County | 2007, 2009, 2012, 2014 | 2012 |
| Low access and no vehicle | USDA Food Access Research Atlas by Census Tract | 2010 | 2010 |
| Crime rate | HRS/CDR Uniform Crime Reports | Every year | 2012 |
| Median Income | USDA Food Environment Atlas by County | 2010, 2015 | 2010 |
| **Health-compromising behaviors** | | | |
| Smoking | HRS Core Interview^a^ | 2010, 2012, 2014, 2016 | 2012 |
| Drinking Alcohol | HRS Core Interview^a^ | 2010, 2012, 2014, 2016 | 2012 |
| Exercise | HRS Core Interview^a^ | 2010, 2012, 2014, 2016 | 2012 |
| Diet | HRS Health Care and Nutrition Mail Study | 2013 | 2012 |
| **Physiological dysregulation** | | | |
|  | HRS Biomarker^b^  HRS Section I Physical Measures^b^ | 2010-2012,  2012-2014,  2014-2016 | 2014-2016 |
| **CVD** | HRS Core Interview | 2010, 2012, 2014, 2016 | 2016 |

Note. ^a^ core interviews were administered every 2 years.

^b^ every 2 years, a random half of study sample was interviewed for collecting biomarker data. Two half samples were combined to get a full sample.

Supplemental Table 3. Reversed serial mediation analysis (obesogenicity🡪 dysfunction 🡪 behaviors 🡪 CVD) using data from 2010 – 2014 (n = 12,317)

| Path | Coefficient | Boot LLCI | Boot ULCI |
| --- | --- | --- | --- |
| Total effects of X on Y | 0.0911 | 0.0487 | 0.1345 |
| - Direct effects of X on Y | 0.0849 | 0.0414 | 0.1284 |
| - Indirect effects of X on Y | 0.0071 | 0.0042 | 0.0111 |
| *through dysfunction* | 0.0046 | 0.0023 | 0.0079 |
| *through behaviors* | 0.0023 | 0.0008 | 0.0047 |
| *serially through dysfunction and behaviors* | 0.0002 | 0.0001 | 0.0004 |
| Effects through mediators |  |  |  |
| - a_1_ (X→ dysfunction) | 0.0380 | 0.0196 | 0.0565 |
| - a_2_ (X→ behaviors) | 0.0288 | 0.0104 | 0.0472 |
| - b_1_ (dysfunction →Y) | 0.1215 | 0.0796 | 0.1633 |
| - b_2_ (behaviors →Y) | 0.0799 | 0.0377 | 0.1222 |
| - dysfunction → behaviors | 0.0680 | 0.0504 | 0.0856 |

Notes.

Age, sex, highest degree, race/ethnicity, county-level education, and county-level population density were adjusted in the analysis.

X, environmental obesogenicity

Y, presence of self-reported CVD

X, dysfunction, and behaviors were standardized prior to analysis. Coefficients presented are partially standardized log regression coefficients, except for a_1_ and a_2_, which are partially standardized regression coefficients.

Boot LLCI, bootstrapped lower limit confidence interval

Boot ULCI, bootstrapped upper limit confidence interval
